# Color Matters: A Study Exploring the Influence of Packaging Colors on University Students’ Perceptions and Willingness to Pay for Organic Pasta

**DOI:** 10.3390/foods13193112

**Published:** 2024-09-29

**Authors:** László Bendegúz Nagy, Ágoston Temesi

**Affiliations:** Department of Agricultural Business and Economics, Institute of Agricultural and Food Economics, Hungarian University of Agriculture and Life Sciences, Villányi Str. 29-43, 1118 Budapest, Hungary; temesi.agoston@uni.mate.hu

**Keywords:** pasta, experimental auction, laboratory experiment, sustainability, trust

## Abstract

The organic food market’s rapid expansion necessitates an understanding of factors influencing consumer behavior. This paper investigates the impact of packaging colors on perceptions and willingness to pay (WTP) for organic foods, utilizing an experimental auction among university students. Drawing on previous research, we explore how colors influence perceived healthiness, premiumness, trust, and sustainability. The results indicate nuanced responses to different colors, emphasizing the need for businesses to adopt tailored packaging strategies. White and green dominate organic food packaging, aligning with associations of freshness and health. However, the study uncovers varied consumer responses, suggesting a more intricate relationship between color, trust, premiumness, and healthiness perceptions. Demographic factors such as age, gender, income, and residence areas influence WTP for organic foods with different colors, emphasizing the importance of diverse consumer segments in marketing strategies. Trust and perceived premiumness significantly influence WTP, highlighting their pivotal role in consumer valuation. The results highlight that green packaging builds trust among non-organic buyers, while organic buyers are influenced by a broader range of colors that emphasize premiumness and healthiness. The study concludes that businesses in the organic food market should carefully consider color choices in branding and packaging to effectively communicate product qualities and align with consumer values.

## 1. Introduction

The packaging of food products plays a critical role in consumer perception and purchasing behavior. As the market becomes increasingly competitive, the visual appeal of packaging, including color, has emerged as a significant factor influencing consumer decisions. This research aims to explore the willingness to pay (WTP) for organic pasta packaged in different colored labels—white, blue, green, and black—while also examining the perceived premiumness, healthiness, trust, and sustainability associated with each color.

Color psychology suggests that colors can evoke specific emotions and associations, impacting consumer preferences and behavior. For instance, white is often associated with purity and simplicity, blue with trust and reliability, green with health and sustainability, and black with sophistication and luxury [1]. These associations are crucial in the context of food packaging, where visual cues can significantly influence consumer perceptions of a product’s quality, safety, and value [2]. The color of food packaging has been identified as a significant factor affecting consumer perceptions and preferences. Clydesdale [3] emphasizes that the color of a product is among its most crucial attributes, and this holds true for food items. Marozzo et al. [4] note that colors are capable of conveying messages, and this ability can significantly influence consumer behavior. Wang and Chang [5] suggest that the color of packaging can affect the taste perception of a product. The evidence that color can influence health judgments is significant. Steiner and Florack [6] assert that people use color as an explicit cue to make a judgment, applying declarative knowledge about the meaning of color cues.

Understanding how color affects WTP is vital for marketers and product developers. Willingness to pay is not merely a reflection of a product’s intrinsic value but also of the perceived added value derived from its packaging. Previous studies have indicated that consumers are willing to pay more for products that they perceive as premium, healthy, trustworthy, and sustainable. For instance, the growing interest in organic foods is driven by the broader societal shift towards healthier and more sustainable lifestyles [7,8,9]. Consumers have become more discerning, not only considering the nutritional aspects of food but also examining the overall quality, authenticity, and sustainability of the products they buy.

Examining the packaging colors of organic foods in the market reveals certain trends. White and green are dominant colors, aligning with the association of these colors with freshness, health, and organic qualities. Blue, yellow, and red colors are also present, albeit less frequently [10]. This observation raises questions about consumer perceptions and associations with different colors in the context of organic foods. The colors used in the presentation, packaging, and branding of organic food products can influence consumers’ overall perception of the product’s quality, authenticity, and sustainability [11]. Consumers are increasingly conscious of the environmental impact of their choices and seek products that align with their values [9].

The perceived premiumness of a product often drives consumers to pay a higher price. Packaging that conveys luxury and exclusivity can create a sense of higher quality, even if the product itself remains unchanged. Black packaging, for example, is traditionally associated with premium products, potentially leading consumers to associate black-labeled products with higher quality and greater value [12,13,14]. However, it is important to note that the color black may also have implications for perceptions of healthiness, as suggested by Karnal et al. [15]. Red labels, for example, lead to longer response time in decision-making than blue labels [16]. Respondents consider more attributes on red labels than on blue labels. Additionally, color affects WTP estimates for certain attributes listed on food labels [16].

Healthiness perception is another critical factor influencing consumer behavior. In an era where health consciousness is on the rise, packaging that suggests a product is healthy can significantly impact purchasing decisions. Green is universally associated with health and nature, which may lead consumers to perceive green-labeled products as a healthier option compared to other colors. This perception can drive an increased WTP as consumers prioritize health-related attributes in their purchasing decisions [17]. Research indicates that green and earthy tones are often associated with the organic nature and environmental friendliness of a product [10]. Hallez et al. [18] add nuance to this understanding, suggesting that cooler colors, such as blue and green, can influence perceptions of healthiness and sustainability. Green labels, for instance, increase perceived healthfulness, especially among consumers who place high importance on healthy eating [17]. Conversely, the results of the study by Huang and Lu [19] suggest that red packages are associated with the perception of less healthy content compared to blue packages. Green packaging attempts to suggest the quality of a product, whether it is good to consume or good for the environment. It is also a sign that the product is healthier and has less fat and possibly fewer calories [19].

Trust in a product and its brand is fundamental to consumer decision-making. Packaging colors that convey trustworthiness can enhance consumer confidence in the product. Green is widely recognized for its ability to evoke a sense of trust and dependability. Therefore, green-labeled products may be perceived as a more trustworthy choice, potentially increasing consumers’ WTP due to the enhanced confidence in the product’s safety and reliability [18].

Sustainability is becoming an increasingly important factor in consumer choices, with more individuals seeking environmentally friendly products. Packaging colors that suggest sustainability, such as green, can influence consumer perceptions and behaviors. Consumers may associate green-labeled food with eco-friendliness and sustainable practices, aligning with their values and motivating them to pay a premium for such products [20]. The promotion of green packaging can increase consumers’ perceived value and decrease their perceived risk of green products, thereby directly or indirectly increasing consumers’ purchase intention [21]. Research shows that green packaging is positively influenced by the perceived value and satisfaction with green packaging, while it is negatively affected by perceived risk. Moreover, green loyalty can significantly moderate the relationship between green purchase intention and other related constructs [20].

Color serves as a crucial informational cue for consumers, allowing marketers to influence consumer perceptions through strategic color choices. Su and Wang [22] recommend that if a food is positioned as a vice food, warm-colored packaging is advisable; conversely, if the food is positioned as a virtue product, cool-colored packaging is preferable. Consumers possessing a positive attitude toward visual packaging tend to evaluate the product and brand positively [23]. Green purchase intention is influenced by green packaging via several routes, indicating that perceived value strongly depends on the impression of the packaging [20]. Generally, colors seem to help differentiate between the attractiveness of products, and attractiveness itself is positively related to both healthiness and tastiness [24].

Packaging color properties varied in hue, brightness, and saturation levels. Warmer, saturated, less bright packages enhance sensory expectation and perception. However, warmer, saturated, less bright packages are more attractive and less healthy [25]. Certain colors have a greater potential to arouse associations with a healthful product, while others evoke quite the opposite associations [26]. Thus, colors can strengthen, complement, or undermine expert messages. Wąsowicz et al. [26] found that labels showing blue and yellow colors evoked positive emotions and associations with health and naturalness, while label colors green, amber, and red were positively received as associated with healthful fruit and vegetables. Participants tend to rely on package color for health content inference, but color significantly influenced the healthiness perception specifically for utilitarian products and not for hedonic products [19].

Given the ambiguity in the meaning of color in foods and beverages, it can sometimes be important that the name and description of a food or beverage set the right sensory or hedonic expectations or else help to disambiguate between the different possible meanings that may be associated with a given color [27]. Certain colors have a greater potential to arouse associations with a healthful product, while others evoke quite the opposite associations. Therefore, colors can strengthen, complement, or undermine expert messages [26].

Research also indicates that the perception of green packaging can increase the market share of greenwashed food [21]. However, sustainability marketing rarely meets expectations when an inconsistent color is used [28]. Examining how consumers learn color meanings reveals that these meanings are often transported through social learning and communication. Consumers tend to learn color meanings when colors co-occur with health perceptions or health information [6].

This research aims to provide a comprehensive analysis of how different packaging colors—white, blue, green, and black—influence consumers’ WTP for organic pasta, alongside their perceptions of premiumness, healthiness, trust, and sustainability. By understanding these dynamics, marketers can better tailor their packaging strategies to align with consumer preferences and enhance product appeal [23]. This study will contribute to the existing literature on consumer behavior and packaging design, offering practical implications for the food industry in leveraging color to optimize product positioning and pricing.

The following research questions were developed:

RQ1: How does the color of the packaging influence the willingness to pay for organic foods?
RQ2: How do different packaging colors affect the perceived healthiness, premiumness, trust, and sustainability of organic foods?

## 2. Materials and Methods

A laboratory experiment was set up to investigate how different packaging colors impact consumers’ willingness to pay (WTP) for four products, with pasta chosen as the experimental item due to its widespread use in households. The Becker–DeGroot–Marschak (BDM) experimental auction method [29] was employed to elicit WTP. This method allows researchers to observe and measure consumer behavior in a controlled environment, providing insights into the factors that drive purchasing decisions [30]. The experiment took place on the Buda Campus of the Hungarian University of Agriculture and Life Sciences in Budapest, Hungary, between 9 and 10 October 2023.

**Figure 1:**
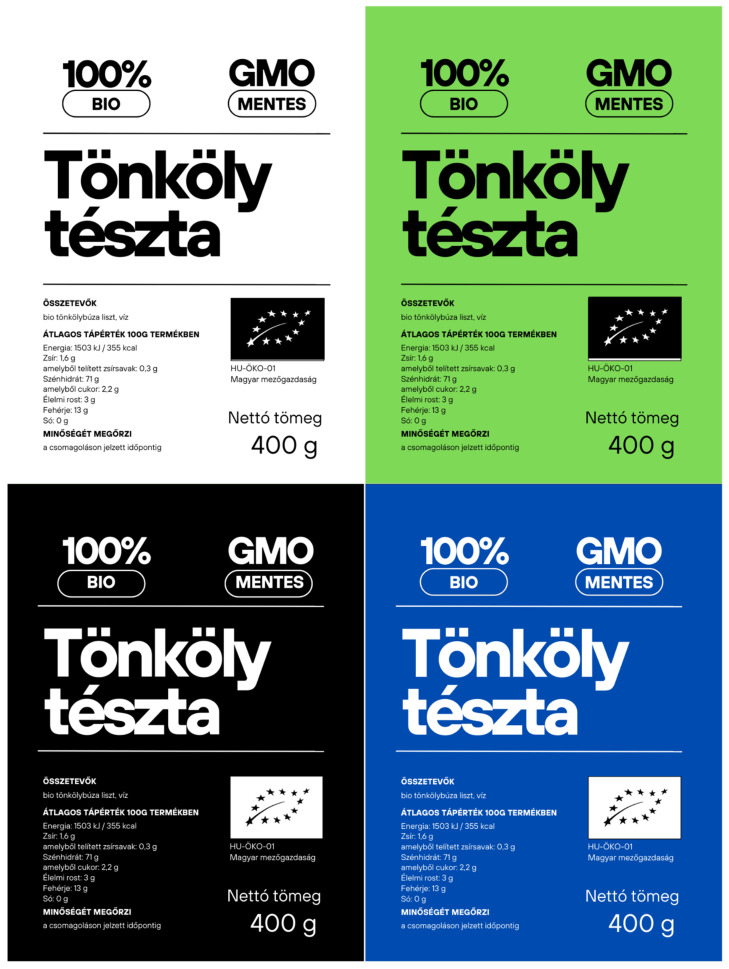
Labels used in the experimental auction. All information and layout on the labels are the same, only background color is different.

Participants were randomly selected on the university campus. Mainly, university students were recruited, as previous research indicates that there is no notable distinction in bidding behavior between participants who are students and those who are not [31]. They were seated in groups of 12–18 individuals, where the experimental method was explained in detail. To ensure understanding, a trial run was conducted using chocolate bars before proceeding with the actual experiment. The four products, presented in a randomized order, shared a similar appearance and package size (400 g) to minimize bias, differing only in the background color of the labels (see Figure 1).

The following color codes were used for the labels: white RGB(255, 255, 255), Hex #FFFFFF; green RGB(126, 217, 87), Hex #7ED957; black RGB(0, 0, 0), Hex #000000; blue RGB(0, 75, 175), Hex #004BAF.

In addition to indicating WTP values and basic demographic details, participants were asked to evaluate the perceived credibility, quality, healthiness, and sustainability of each product. Participants’ attitudes toward responsible food consumption were measured using a 5-question scale on food responsibility developed by Brunsø et al. [32], as responsible attitudes could influence organic consumption. The interest in health and natural products was gauged using scales developed by Roininen et al. [33], considering its potential correlation with the evaluation of organic and functional foods. The frequency of purchasing organic food was also assessed based on Zander et al. [34].

Following the BDM mechanism, a product and a price were randomly drawn from an urn after participants completed the survey. Prices ranged between HUF 250 and 600 (EUR 0.65 and EUR 1.55) in HUF 50 (approximately 12 cents) increments. If the participant’s WTP for the drawn product exceeded the randomly selected price, they were required to purchase the product; otherwise, no transaction occurred.

Overall, 7 experimental rounds were conducted, taking 15–20 min each, with participants receiving oral BDM information based on a written script. The products were displayed to the participants in randomized order across groups to avoid learning effects. The survey was conducted online, and participants independently completed it. Participants did not receive any reward for taking part in the experiment. The study was registered on Aspredicted.org (number 146002) and obtained ethical approval from the Interim Ethical Committee of the Doctoral School of Economic and Regional Sciences (case number 13/2023). Every respondent provided informed consent before participating. Data analysis was carried out using Stata version 17.0 and IBM SPSS 29.0.

We employed the Kolmogorov–Smirnov test to assess the normality of our data distributions. Independent samples *t*-tests were used to compare mean willingness to pay (WTP) and consumer perceptions across different packaging colors. To ensure the reliability of our regression models, we applied the Breusch–Pagan test to check for heteroscedasticity.

## 3. Results

In Table 1, the characteristics of the 102 participants are outlined, providing insights into their demographic distribution, educational background, perceived income, and buying frequency of organic food.

The majority of participants were females (69%), primarily belonging to the 18–25 age group (97%). Residence areas were diverse, with 43% residing in the capital city, 36% in a city or town, and 21% in a village. Educational background predominantly included high school graduates (91%). Participants reported varied perceived incomes, with 11% indicating low, 49% average, and 40% high income levels. Regarding organic food buying frequency, 31% reported never/almost never, 34% less than once per month, 25% 1–2 times per month, and smaller percentages for more frequent purchasing, which corresponds with the organic food consumption habits of the Hungarian population [35].

The participants’ demographics, reflecting varied income levels and organic food buying frequency, offer a crucial context for interpreting the willingness-to-pay (WTP) values. These factors may intersect with color preferences, providing insights into how diverse profiles influence consumer behavior in the experimental auction study.

Table 2 presents the participants’ perceptions of various attributes associated with organic pasta products labeled in different colors. The scale ranges from 1 to 7, with higher values indicating stronger agreement or perception.

Participants expressed a high level of trust across all color-labeled organic pasta products, with mean scores ranging from 5.05 to 5.21. The trustworthiness of the products appeared consistently strong regardless of the color. Sustainability perceptions were generally positive, with mean scores ranging from 4.61 to 4.70. Participants perceived the organic pasta products, regardless of color, as having a commitment to sustainable practices. Perceived premiumness varied across colors, with the highest mean score of 4.92 for black-labeled organic pasta. The white and green color labels also received positive evaluations, scoring 4.33 and 4.42, respectively. Blue-labeled organic pasta had a slightly lower score of 4.44. Healthiness perceptions were consistently high across all color-labeled organic pasta products, with mean scores ranging from 5.04 to 5.21. Participants perceived the products as being notably healthy, irrespective of the color label.

Table 3 displays the participants’ WTP values for organic pasta products with different color labels, along with standard deviation, minimum, and maximum values.

Participants exhibited varying WTP across different colors, with mean values of HUF 544.58/EUR 1.36 (white), HUF 570.87/EUR 1.44 (black), HUF 543.59/EUR 1.35 (green), and HUF 538.71/EUR 1.34 (blue). Standard deviations ranged from 185.76 to 194.41, reflecting the dispersion of WTP values. Notably, all color-labeled products had a potential maximum WTP of 1000.

**Figure 2:**
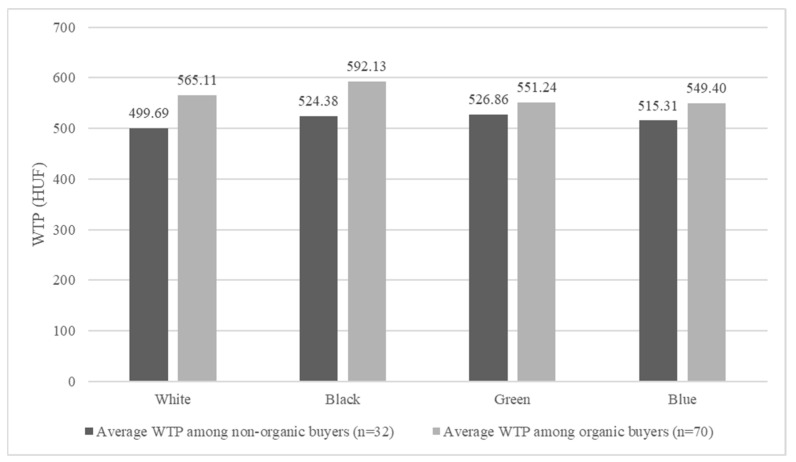
WTP values of non-organic and organic buyers.

To enhance the understanding of different consumer groups, the willingness to pay (WTP) values of participants who never buy organic food (non-organic buyers) and those who purchase organic food to varying degrees (organic buyers) were separately analyzed using *t*-tests. As shown in Figure 2, organic buyers exhibited higher average WTP values compared to non-organic buyers. The most significant difference was observed for the white-labeled product (t = −1.665, *p* = 0.099), while the smallest difference, less than 5%, was noted for the green-labeled product (t = −0.591, *p* = 0.556).

Table 4 provides a detailed insight into the factors influencing participants’ WTP for organic pasta products with different color labels. The drivers include age, gender, income, place of living, organic purchase frequency, trust, sustainability, premiumness, healthiness, price consciousness, quality consciousness, general health interest, natural product interest, and food responsibility. The regression coefficients represent the strength and direction of the influence of each driver on WTP.

Age positively influenced WTP for all colors, with the highest impact observed for white-labeled organic pasta. Gender displayed mixed effects, with females showing a positive influence on WTP for white and blue labels but a negative influence on green-labeled organic pasta. Income had a limited impact, with only white-labeled organic pasta showing a positive association with higher income. Participants residing in the capital city exhibited lower WTP for all colors compared to other residence areas. Higher organic purchase frequency positively correlated with increased WTP for all colors.

Trust, premiumness, and healthiness significantly influenced WTP across all color-labeled organic pasta products, except for green color products, where healthiness was not influencing. Trust and premiumness had particularly strong positive effects, suggesting that these attributes played a pivotal role in participants’ valuation of the products. Price consciousness positively influenced WTP for black-labeled organic pasta, while quality consciousness positively affected WTP for white-labeled organic pasta. General health interest positively affected WTP for green-labeled organic pasta, whereas interest in natural products does not influence WTP significantly. Food responsibility had a mixed impact on WTP, with a negative influence on black-labeled organic pasta and a positive influence on blue-labeled organic pasta.

Similarly to the WTP values, preference drivers were analyzed separately for non-organic and organic buyers. Table 5 displays significant drivers among non-organic and organic participants.

The analysis of preference drivers for non-organic and organic buyers reveals distinct differences in their WTP for the different products. Among non-organic buyers, a significant driver is trust, particularly for the green-labeled product. In contrast, organic buyers showed a broader range of significant drivers. Trust and price consciousness were significant for multiple labels, with trust having substantial effects on the white, black, and blue labels and price consciousness on all the labels. Premiumness was a highly significant driver across all the labels for organic buyers.

## 4. Discussion

The results of our experimental auction study shed light on the intricate relationship between packaging color, consumer perceptions, and willingness to pay (WTP) for organic food products. As the organic food market continues to grow [36], businesses must strategically consider the impact of packaging colors on consumer behavior to effectively cater to evolving preferences and values.

The dominance of white and green colors in organic food packaging aligns with their associations with freshness, health, and organic qualities. These color choices reflect a conscious effort by businesses to appeal to consumers seeking environmentally friendly and nutritious options. Our findings indicate that green is significantly perceived as more sustainable and credible, reinforcing the association of green with the organic nature and environmental friendliness of a product, consistent with prior research [10]. Moreover, green and white colors are significantly considered to be healthier than other colors, which further aligns with consumer expectations for organic and healthful products. Cooler colors like blue and green influence perceptions of healthiness and sustainability, supporting the findings of Hallez et al. [18].

The study highlights notable differences between organic and non-organic buyers in their perceptions and willingness to pay (WTP) for organic pasta products with various color labels. For non-organic buyers, green packaging is particularly effective in building trust as they associate green with trustworthiness, making it a key color for appealing to their preferences. In contrast, organic buyers exhibit a more complex set of preferences where trust is just one of several influential factors.

Organic buyers show significant sensitivity to other product attributes, such as premiumness and healthiness, which also drive their WTP. For these consumers, packaging colors can be more diverse, with white, black, and blue also being effective when these attributes are highlighted. Trust remains important but is complemented by a strong emphasis on the perceived quality and health benefits of the product.

Our study supports the notion that packaging colors go beyond aesthetic appeal; they play a significant role in shaping consumers’ trust, perceptions of premiumness, and evaluations of healthiness. Black packaging, for example, is significantly considered premium compared to other colors, which aligns with the findings of Pereira [12] and Klimchuk & Krasovec [13]. However, the nuanced impact of black packaging on healthiness perceptions, as suggested by Karnal et al. [15], underscores the complexity of color associations.

The analysis of demographic factors reveals intriguing patterns in WTP across different colors. Age, gender, income, and residence areas exhibit varying degrees of influence, emphasizing the importance of considering diverse consumer segments in marketing strategies. For instance, the positive influence of higher organic purchase frequency on WTP for all colors suggests a potential market segment that values and is willing to invest in organic products. Moreover, the significant impact of trust and premiumness on WTP underscores the pivotal role these attributes play in consumer valuation. Businesses should prioritize building trust and conveying a sense of premium quality in their organic food products to enhance their market competitiveness.

## 5. Conclusions

The organic food market’s growth is indicative of the increasing consumer demand for healthier and more sustainable food options. The colors used in the presentation, packaging, and branding of organic food products play a significant role in shaping consumer perceptions and preferences. The experimental auction method provides a valuable avenue for researchers to explore the complex interplay of cognitive and economic factors that influence consumers’ willingness to pay for organic foods based on color-related perceptions. Businesses in this market must consider the implications of color choices in their branding and packaging strategies to align with consumer values and effectively communicate the qualities of their products.

The study reveals distinct differences between organic and non-organic buyers regarding their perceptions and willingness to pay (WTP) for pasta products with various color labels. While green packaging builds trust among non-organic buyers, trust remains a significant factor for organic buyers across a wider range of colors, including white and black. Additionally, for organic buyers, packaging color can be varied based on other product attributes, such as premiumness and healthiness, which are crucial drivers of WTP. These insights suggest that while green packaging may effectively build trust among non-organic consumers, a more nuanced approach, emphasizing premiumness and healthiness in the packaging design, could better cater to organic buyers. This tailored strategy could enhance consumer satisfaction and willingness to pay in both segments.

Organic producers should carefully consider packaging color choices based on their target market and objectives. For emphasizing healthiness and sustainability, white and green are suitable, while black may appeal to those emphasizing premium quality. The nuances in consumer responses to colors highlight the need for a tailored approach, ensuring alignment with the values and preferences of diverse consumer segments in the dynamic organic food market.

In conclusion, the findings provide actionable insights for businesses aiming to navigate the dynamic landscape of consumer preferences in the organic food market. However, it is important to note that these results are preliminary and are valid only for a small section of the population—namely, university students in Hungary. To confirm the robustness and applicability of these insights, further studies need to be conducted with a broader and more diverse population. This would help validate the conclusions and ensure they reflect wider consumer behaviors across different demographics and cultural contexts.

## 6. Limitations

While this study contributes valuable insights, it is not without limitations. This study was conducted with a relatively small sample size, consisting solely of university students from a single institution in Hungary. As a result, the findings are only applicable to a narrow segment of the population: university students in Hungary. The specific demographic and cultural context may limit the generalizability of the results to other populations or regions. Additionally, the study’s controlled environment may not fully capture real-world consumer behavior. Also, the brightness and saturation levels of the different colors were not taken into account in this particular research. To enhance the validity and broader applicability of these findings, future research should replicate this study across different cultural contexts and include a more diverse range of population demographics. This would allow for a deeper understanding of consumer behavior and willingness to pay across varied groups and settings.

## Figures and Tables

**Table 1:** Characteristics of participants (n = 102).

**Sex at Birth**	**n**	**%**
Male	32	31%
Female	70	69%
**Age group**		
18–25	99	97%
26–35	3	3%
**Residence area**		
Capital city	44	43%
City/town	37	36%
Village	21	21%
**Education**		
High school	93	91%
Diploma	9	9%
**Perceived income**		
Low	11	11%
Average	50	49%
High	41	40%
**Organic food buying frequency**		
Never/almost never	32	31%
Less than once per month	35	34%
1–2 times per month	25	25%
Once per week	7	7%
Several times per week	3	3%

**Table 2:** Perceived trust, sustainability, premiumness, and healthiness of the different color packaging.

	Trust	Sustainability	Premiumness	Healthiness
White	5.08	4.64	4.33	5.21
Black	5.05	4.61	4.92	5.15
Green	5.21	4.70	4.42	5.20
Blue	5.07	4.61	4.44	5.04

Note: Perceived trust, sustainability, premiumness, and healthiness values are statistically different according to Kolmogorov–Smirnov tests (at *p* < 0.001).

**Table 3:** WTP values of the different color-labeled organic pasta products.

	WTP	Std. Dev.	Min	Max
White	544.58	185.76	0	1000
Black	570.87	198.04	0	1000
Green	543.59	192.5	0	1000
Blue	538.71	194.41	0	1000

Note: values are displayed in Hungarian Forint (HUF). HUF 1 is appr. EUR 0.0025. WTP values are statistically different according to Kolmogorov–Smirnov tests (at *p* < 0.001).

**Table 4:** Preference drivers.

	White WTP	Black WTP	Green WTP	Blue WTP
Age	0.98	0.98	0.16	0.92
Gender	0.92	−0.10	−0.98	0.23
Education	−0.46	−0.81	0.19	−0.62
Income	0.09	−0.28	0.39	0.32
Place of living	−0.61	−0.56	−0.08	−0.16
Organic purchase	0.82	1.35	0.71	0.34
Trust	2.74 **	3.43 **	2.49 **	2.73 **
Sustainability	0.24	0.61	0.48	1.06
Premiumness	4.17 **	2.73 **	2.21 **	2.64 **
Healthiness	2.03 **	2.27 **	1.16	2.35 **
Price consciousness	1.47	2.02 **	1.28	1.12
Quality consciousness	1.61	0.89	−0.33	0.75
General health interest	0.23	0.01	1.79 *	1.02
Natural product interest	−0.74	0.34	−0.24	−1.05
Food responsibility	−0.24	−0.84	−0.58	0.77
Constant	−1.08	−0.66	−0.21	−0.86
R2	0.161	0.271	0.195	0.261
Chi2	76.53	106.30	53.62	94.16
*p*	0.000	0.000	0.000	0.000

Note: * *p* < 0.1; ** *p* < 0.05; Breusch–Pagan test of independence: chi^2^(6) = 375.985, Pr = 0.0000.

**Table 5:** Preference drivers of non-organic and organic buyers.

	White WTP	Black WTP	Green WTP	Blue WTP
Non-organic buyers ^1^ (n = 32)				
Trust	2.00 **	2.76 **	3.00 **	2.79 **
Sustainability	0.09	0.16	−1.28	−0.07
Premiumness	2.01 **	1.49	1.45	1.39
Healthiness	0.05	0.02	1.80 *	0.89
Price consciousness	−0.45	−0.66	0.05	−1.40
Constant	1.46	1.49	0.52	1.17
R^2^	−0.069	0.157	0.066	0.372
Chi^2^	15.53	36.94	31.32	45.16
*p*	0.008	0.000	0.000	0.000
Organic buyers ^2^ (n = 70)				
Trust	3.52 **	3.11 **	1.51	2.97 **
Sustainability	−0.05	0.57	0.14	0.74
Premiumness	4.23 **	2.66 **	2.38 **	3.86 **
Healthiness	1.22	1.33	0.30	0.70
Price consciousness	1.92 *	2.49 **	1.81 *	2.22 **
Constant	−0.13	−0.64	0.41	−0.11
R^2^	0.207	0.325	0.174	0.221
Chi^2^	67.62	63.89	24.75	70.32
*p*	0.000	0.000	0.000	0.000

Note: * *p* < 0.1; ** *p* < 0.05; ^1^ Breusch–Pagan test of independence: chi2(6) = 96.823, Pr = 0.0000; ^2^ Breusch–Pagan test of independence: chi2(6) = 241.929, Pr = 0.0000.

## Data Availability

The original contributions presented in the study are included in the article, further inquiries can be directed to the corresponding author.
